# Interpretable Spectral Features for Cross-Session EEG Biometric Identification and Verification

**DOI:** 10.3390/s26154811

**Published:** 2026-07-29

**Authors:** Cai Chen, Jiazheng Sun, Danyang Lv, Chongxuan Tian, Shuxian Li, Ningling Zhang, Tao Jing

**Affiliations:** 1 Shandong Institute of Advanced Technology, Chinese Academy of Sciences, Jinan 250100, China; d202582315@hust.edu.cn (C.C.); jz.sun@sdiat.ac.cn (J.S.); dy.lv@sdiat.ac.cn (D.L.); 2 State Key Laboratory of Environment Health (Incubation), School of Public Health, Tongji Medical College, Huazhong University of Science and Technology, #13 Hangkong Road, Wuhan 430030, China; m202575905@hust.edu.cn; 3 Key Laboratory of Environment and Health, Ministry of Education, School of Public Health, Tongji Medical College, Huazhong University of Science and Technology, #13 Hangkong Road, Wuhan 430030, China; 4 Key Laboratory of Environment and Health (Wuhan), Ministry of Environmental Protection, School of Public Health, Tongji Medical College, Huazhong University of Science and Technology, #13 Hangkong Road, Wuhan 430030, China; 5 School of Control Science and Engineering, Shandong University, Jinan 250100, China; 202420777@mail.sdu.edu.cn

**Keywords:** EEG sensing, biometric sensing, cross-session authentication, biometric verification, calibration, wearable sensors

## Abstract

Electroencephalogram (EEG)-based biometric sensing provides a promising pathway for secure and user-specific human authentication, but practical deployment remains limited by cross-session non-stationarity and potentially optimistic evaluation protocols caused by window- or event-level leakage. This study developed a cross-session EEG biometric sensing framework using interpretable spectral features and target-session calibration, with trial-level non-overlapping data partitioning to prevent overlap between training, calibration, and blind-test sets. Multi-channel EEG events were segmented into non-overlapping 2 s windows and aggregated into event-level samples to ensure strict isolation among training, validation, calibration, and blind testing subsets. Power spectral density, differential entropy, and log-variance features were evaluated using support vector machine, linear discriminant analysis, logistic regression, random forest, and a compact convolutional neural network designed for EEG decoding (EEGNet). Both closed-set identification and biometric verification were assessed using Rank-*N* accuracy, cumulative match characteristic curves, macro-F1, equal error rate, area under the curve, and true acceptance rate (TAR) at fixed false acceptance rate (FAR) levels. In the in-house cross-day dataset, limited target-session calibration improved Rank-1 accuracy from 44.82% to 98.70% for support vector machine (SVM) and from 19.93% to 99.94% for linear discriminant analysis (LDA). SVM with differential entropy achieved an equal error rate (EER) of 1.04 ± 0.60%, area under the receiver operating characteristic curve (AUC) of 0.9985 ± 0.0008, and TAR@FAR = 0.1% of 98.44 ± 1.06%. External validation on the public multi-session motor imagery dataset confirmed the calibration benefit. These results demonstrate strong closed-set identification and score-level verification performance under a cohort-wide, calibration-assisted cross-session experimental protocol.

## 1. Introduction

Electroencephalography (EEG) has attracted increasing attention as a biometric modality for person identification and authentication. Conventional biometric traits, such as fingerprints, facial images, iris patterns, and voice, have been widely used in access control and security systems. However, these external traits can be observed, copied, spoofed, or permanently compromised once the biometric template is leaked. In contrast, EEG signals are generated by internal neural activity and have several attractive properties for biometric applications, including endogenous neural origin and task-dependent physiological characteristics [1,2]. These characteristics make EEG a promising complement to conventional biometrics, particularly for high-security authentication, wearable monitoring, medical device binding, and brain–computer interface (BCI)-based interaction systems.

Existing EEG biometric studies have demonstrated that neural signals contain subject-specific information in spectral power [3], temporal dynamics [4], evoked responses [5], and brain network organization [6]. Various EEG paradigms have been explored, including resting-state EEG [5], event-related potentials [7], steady-state visual evoked potentials [8], motor imagery [5], auditory evoked responses [9], and affective EEG [10,11]. In parallel, recognition algorithms have evolved from handcrafted spectral and statistical features to deep representation learning models, including convolutional neural networks [12], recurrent neural networks [13], Transformers [1], and graph neural networks [11]. These studies have shown that EEG contains discriminative individual signatures and can be used for both identification and authentication. Despite these advances, practical EEG biometrics remain challenging. Many studies report high performance under within-session or randomly shuffled evaluation protocols [1,3]. Such protocols may overestimate real-world reliability because training and testing samples can share session-specific noise, electrode contact conditions, temporal dependencies, and task-state characteristics. For deployable biometric systems, the key question is not only whether a classifier can separate subjects under controlled within-session conditions, but whether the extracted identity representation remains stable when the same user returns on another day.

Cross-day and cross-session variability is therefore one of the central problems in EEG biometrics. EEG signals are non-stationary and can be influenced by electrode placement, impedance changes, fatigue, attention, mental state, and environmental noise. A model trained on one recording session may fail when applied to a later session. Recent studies have increasingly investigated cross-session EEG biometric recognition using domain adaptation, attention-based modeling, graph representations, and session-invariant feature learning [14,15,16]. However, there remains a need for strict, transparent, and reproducible protocols that prevent event-level or window-level leakage and evaluate both identification and verification performance. Motor imagery (MI) EEG provides a useful paradigm for studying cross-session EEG identity recognition. MI is one of the most widely used BCI paradigms and produces structured task-related EEG responses during imagined limb movements. Compared with resting-state EEG, MI trials provide clearer task organization and are closer to practical BCI interaction scenarios. At the same time, MI EEG is highly variable across sessions, making it a challenging testbed for evaluating whether subject-specific EEG signatures remain identifiable across days. In this study, we focus on cross-day/session EEG identity recognition using motor-related EEG events and investigate whether limited target-session calibration can compensate for session-related distribution shifts.

To address these issues, we propose a lightweight and interpretable EEG biometric signal processing framework based on handcrafted spectral and statistical features. Specifically, relative power spectral density (PSD), differential entropy (DE), and log-variance features are extracted from multi-channel EEG epochs corresponding to left- and right-hand motor imagery trials to capture subject-specific frequency-domain and signal-complexity characteristics. Classical classifiers, including support vector machine (SVM), linear discriminant analysis (LDA), logistic regression (LR), and random forest (RF), are used to assess these features under two cross-session settings: no-calibration and calibration. An EEGNet-like compact neural network is also included as a raw EEG baseline.

Importantly, the present study evaluates EEG biometrics from both identification and verification perspectives. Closed-set identification accuracy measures whether the system can assign an EEG event to the correct enrolled subject. Practical biometric authentication, however, also requires verification metrics that reflect security-related decision performance. Therefore, in addition to Rank-*N* identification accuracy and cumulative match characteristic (CMC) curves, we report equal error rate (EER), false accept rate (FAR), false reject rate (FRR), receiver operating characteristic (ROC) curves, and true accept rate at fixed FAR levels. This dual evaluation is essential because high multi-class classification accuracy does not necessarily guarantee reliable authentication under low false-acceptance constraints.

The main contributions of this study are fourfold. First, we propose a cross-session EEG biometric sensing protocol based on trial-level non-overlapping data partitioning, in which training, validation, calibration, and blind testing subsets are kept strictly separated. Second, we develop an interpretable sensor-signal processing framework based on PSD, DE, and log-variance features for EEG identity representation. Third, we evaluate the framework from both identification and verification perspectives, including Rank-*N* accuracy, CMC curves, EER, AUC, and TAR at low FAR operating points. Fourth, we analyze calibration burden, reduced-channel feasibility, task robustness, and external generalization on the external public multi-session MI dataset [17], providing empirical evidence to inform the future development of cross-session EEG-based authentication frameworks.

## 2. Materials and Methods

### 2.1. Dataset and Experimental Paradigm

The in-house EEG dataset contained recordings from subjects performing eight motor-related task conditions across two independent recording sessions conducted on different days. These two sessions were acquired separately for each subject and were treated as different temporal recording sessions rather than randomly divided subsets from a single continuous recording. The task conditions were designed around hand laterality, force level, and execution/imagery state. Specifically, the eight task labels corresponded to right-hand grip execution at 1 kg, imagined right-hand grip at 1 kg, left-hand grip execution at 1 kg, imagined left-hand grip at 1 kg, right-hand grip execution at 10 kg, imagined right-hand grip at 10 kg, left-hand grip execution at 10 kg, and imagined left-hand grip at 10 kg. Thus, the paradigm included both motor execution and motor imagery conditions involving left-/right-hand movements and low/high force levels. This study was approved by the Ethics Committee of the Shenzhen Institutes of Advanced Technology, Chinese Academy of Sciences (No. SIAT-IRB-220715-H0602).

Continuous EEG signals were recorded from 64 scalp electrodes at a sampling rate of 500 Hz using the NeuroSCI EEG acquisition system. Each event was associated with a task label and onset latency. Although the original experimental paradigm was designed around motor-related tasks, the present study used these events for EEG-based identity recognition. Subject identity served as the classification target, while task labels were retained for single-task contribution and leave-one-task-out generalization analyses. Therefore, the main problem was formulated as cross-session EEG biometric identification and verification rather than conventional MI task classification. For each subject, the first recording session was used as the source session for training and validation, whereas the second session was used as the target session for calibration and blind testing. This design represents a calibration-assisted cross-session biometric scenario in which labeled target-session samples from all enrolled participants are available for a one-time cohort-wide supervised recalibration before final blind identification or verification. The final in-house dataset included 14 participants, as shown in Table 1, corresponding to a closed-set chance level of 1/14=7.14%. Each participant performed 10 repetitions of each of the eight task conditions in each recording session, yielding 1120 planned events per session. After event screening, 1108 valid events were retained from the source session and 1117 from the target session. Thus, each participant–task stratum generally contained 10 valid target-session events, with only a small number of events excluded during quality control.

Validation was performed on the public multi-session MI dataset [17], which contains EEG recordings from 25 subjects across five independent sessions. This dataset provides a suitable independent benchmark for evaluating whether the proposed framework generalizes beyond a single acquisition protocol. For the external public dataset, Sessions 1–3 were used as source sessions for training and validation, whereas Sessions 4–5 were pooled as target-session data and then divided into calibration and blind testing subsets using the same event-level split protocol. Consequently, calibration and blind testing events could originate from the same target recording session. The public dataset should therefore be interpreted as pooled target-session validation rather than a fully session-blocked evaluation.

### 2.2. Overview of the Proposed Framework

**Figure 1:**
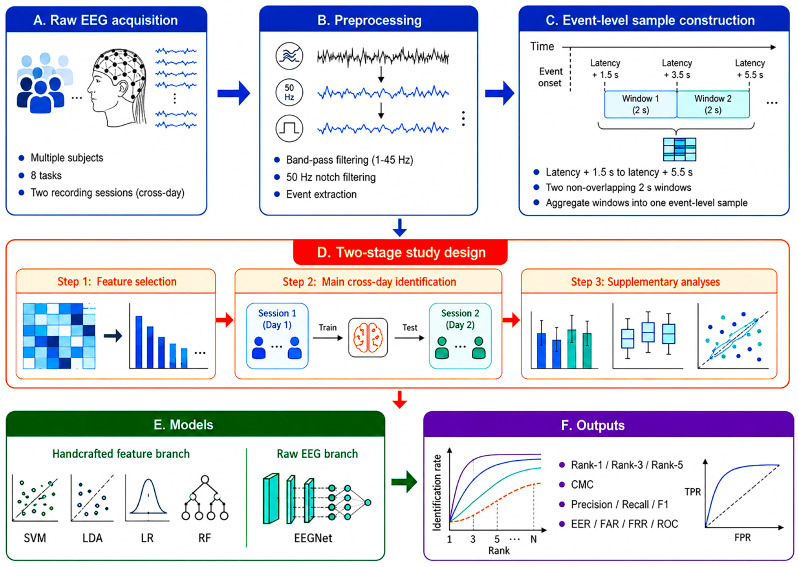
Overall workflow of the proposed cross-day EEG biometric identification and verification framework.

The overall workflow of the proposed cross-session EEG biometric framework is shown in Figure 1. The framework includes raw EEG acquisition, preprocessing, event-level sample construction, cross-session splitting, feature extraction, model training, and biometric evaluation. Two modeling branches were considered. The handcrafted feature branch used spectral and statistical representations followed by classical classifiers. The raw EEG branch used an EEGNet-like compact convolutional network as an end-to-end baseline. The final outputs included both closed-set identification metrics and biometric verification metrics. The analysis pipeline was implemented in Python 3.8 and mainly relied on NumPy, SciPy, scikit-learn, PyTorch, seaborn, and MNE-Python.

### 2.3. Preprocessing and Event-Level Sample Construction

All EEG data were preprocessed before feature extraction and model training. The EEG signals were re-referenced using a common average reference calculated across all 64 scalp channels. No bad-channel interpolation was applied, and all scalp channels were retained for subsequent preprocessing and analysis. Continuous EEG signals were band-pass filtered between 1 and 45 Hz using a zero-phase Butterworth filter to retain the major EEG rhythms while suppressing slow drifts and high-frequency noise. A 50 Hz notch filter was subsequently applied to reduce power-line interference. Infomax independent component analysis was performed separately for each of the 28 recording sessions. Artifact-related components were classified using ICLabel. Components with a predicted probability of at least 0.90 for either the eye or muscle class were removed. Across all recording sessions, 220 independent components were removed, including 143 eye-related components and 77 muscle-related components, corresponding to an average of 7.86 removed components per session. EEG events were extracted according to their latency markers. The experimental design included 2240 planned events, corresponding to 14 participants, eight task conditions, 10 repetitions per condition, and two independent recording sessions. Because the task markers were transmitted to the EEG acquisition system through a serial-port interface, occasional marker-transmission or parsing failures occurred. Of the 15 excluded events, nine were excluded because the corresponding task markers were missing, whereas six were excluded because the recorded labels were malformed, unparseable, or could not be mapped to one of the eight predefined task conditions. These exclusions were based solely on marker integrity and label validity and were applied before dataset splitting, model training, and evaluation. The remaining 2225 valid event-level samples comprised 1108 source-session events and 1117 target-session events. For each valid event, the analysis interval was defined from 1.5 to 5.5 s after the event marker. Within this 4 s interval, two non-overlapping 2 s windows were extracted. Features were first computed independently from the two windows and then averaged to construct one event-level sample.

**Figure 2:**
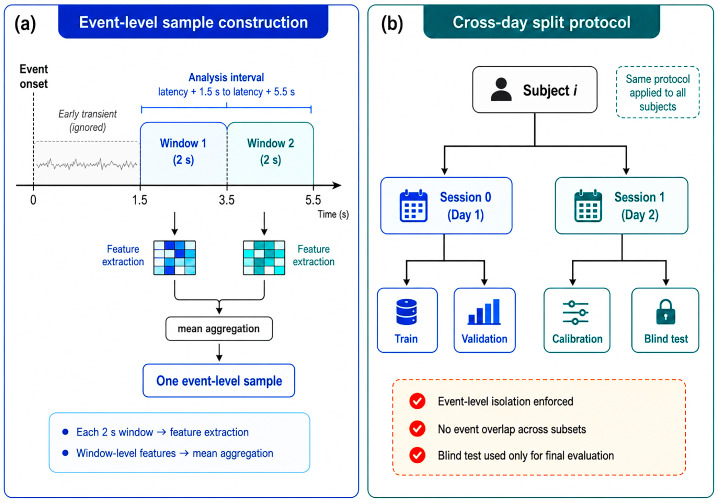
Event-level sample construction and cross-day split protocol.

For each extracted window, channel-wise temporal mean subtraction was applied to reduce constant offsets and session-dependent amplitude shifts. The event-level sample construction strategy is shown in Figure 2a. For each event, the early transient period after event onset was ignored, and the analysis interval was defined from latency + 1.5 s to latency + 5.5 s. Within this 4 s interval, two non-overlapping 2 s windows were extracted. Features were first computed independently from each window and then averaged to form one event-level sample. Let $f_{e,1}$ and $f_{e,2}$ denote the feature vectors extracted from the first and second non-overlapping 2 s windows of event *e*, respectively. The final event-level feature vector was calculated as

(1) $$f_{e}=\frac{f_{e,1}+f_{e,2}}{2},$$

where $f_{e}$ denotes the aggregated feature vector used for subsequent classifier training and evaluation. This design ensured that each marker-defined EEG trial, referred to here as a physical EEG event, contributed only one event-level sample to the downstream identity recognition task. Therefore, different data subsets did not share windows originating from the same event, which is critical for preventing overly optimistic performance estimates caused by window-level leakage. For the external public validation dataset, each trial was treated as one event, and the same windowing and aggregation strategy was applied.

### 2.4. Cross-Session Split and Calibration Settings

The cross-session split protocol is shown in Figure 2b. For each subject, Session 0 was used to construct source-domain training and validation subsets. The validation set was used only for model selection and hyperparameter tuning. Session 1 was used to construct the target-domain calibration and blind testing subsets. The blind testing set was reserved only for final evaluation. Strict event-level isolation was enforced across training, validation, calibration, and blind testing sets. Two evaluation settings were considered. In the non-calibrated setting, models were trained only on source-session training samples and selected using source-session validation samples. The trained model was then directly evaluated on the target-session blind testing set without using target-session calibration data. This setting evaluated direct cross-day generalization. In the calibration setting, a small supervised subset of target-session samples was combined with the source-session training samples, and the classifier was retrained once before blind testing. The remaining target-session samples were kept separate and used only for final blind identification or verification. This setting therefore evaluates session-adaptive biometric recognition rather than authentication without any target-session calibration. Calibration ratios of 0%, 5%, 10%, 15%, and 20% were evaluated to quantify how much target-session information was required to compensate for cross-session distribution shift.

For each repeated run, the target-session calibration and blind testing subsets were generated using a participant–task stratified random split. Specifically, for each participant and each task condition, valid target-session event IDs were randomly shuffled using the run-specific seed. The predefined calibration proportion was assigned to the calibration subset, and the remaining events were reserved for blind testing. Thus, calibration sampling was stratified by both participant identity and task condition. The target-session split was not temporally blocked; therefore, adjacent target-session events could in principle enter different subsets. Accordingly, the revised protocol should be interpreted as preventing event/window overlap across subsets rather than eliminating all possible same-session dependence. Calibration samples were obtained from all enrolled participants included in the closed-set biometric system. These labeled target-session samples were pooled with the source-session training data, and a single multi-class classifier was retrained once before blind testing. Therefore, the present procedure represents cohort-wide supervised recalibration using participant-labeled target-session data, rather than an individual user enrollment update or participant-specific incremental calibration.

### 2.5. Handcrafted Feature Extraction

**Figure 3:**
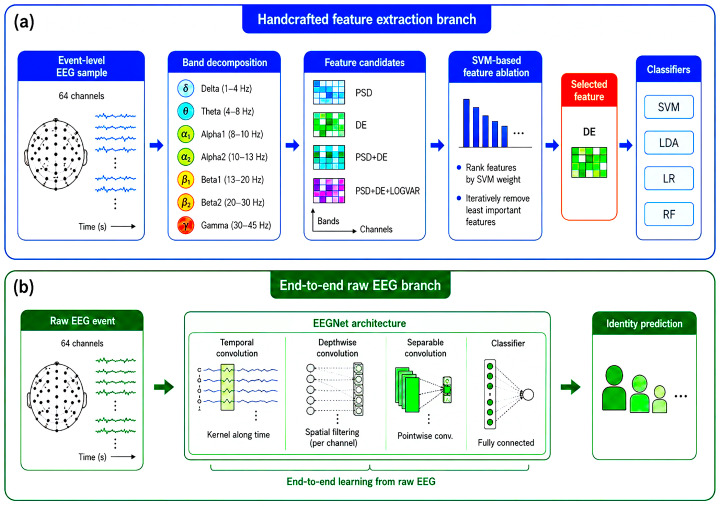
Handcrafted feature extraction and raw EEG modeling branches. (**a**) The handcrafted branch decomposed EEG events into frequency bands and extracted PSD, DE, PSD+DE, and PSD+DE+LOGVAR features. (**b**) The raw EEG branch used an EEGNet-like compact convolutional network.

The handcrafted feature extraction branch is shown in Figure 3a. For each event-level EEG sample, features were extracted from seven frequency bands: delta (1–4 Hz), theta (4–8 Hz), alpha1 (8–10 Hz), alpha2 (10–13 Hz), beta1 (13–20 Hz), beta2 (20–30 Hz), and gamma (30–45 Hz). Three feature types were evaluated, including relative power spectral density (PSD), differential entropy (DE), and log-variance (LOGVAR). The power spectral density of each channel was estimated using Welch’s method. For channel *c* and frequency band *b*, the relative PSD was calculated as

(2) $$rPSD_{c,b}=\frac{P_{c,b}}{\sum_{k=1}^{B}P_{c,k}},$$

where $P_{c,b}$ represents the absolute power of channel *c* in frequency band *b*, and *B* denotes the total number of frequency bands. This normalization reduces inter-session amplitude variation while preserving subject-specific spectral characteristics. Differential entropy was calculated under the assumption that the EEG signal within each frequency band follows a Gaussian distribution. The DE feature for channel *c* and frequency band *b* was defined as

(3) $$DE_{c,b}=\frac{1}{2}\log\left(2\pi e\sigma_{c,b}^{2}\right),$$

where $\sigma_{c,b}^{2}$ represents the variance of the EEG signal of channel *c* within frequency band *b*. DE quantifies the uncertainty and complexity of EEG oscillatory activity and has been widely used for EEG-based representation learning. The log-variance feature was extracted from the time-domain EEG signal of each channel,

(4) $$LOGVAR_{c}=\log\left(\frac{1}{N}\sum_{n=1}^{N}{\left(x_{c,n}-{\bar{x}}_{c}\right)}^{2}\right),$$

where $x_{c,n}$ denotes the *n*-th sample of channel *c*, ${\bar{x}}_{c}$ represents the temporal mean, and *N* is the number of time points in the EEG segment. LOGVAR captures channel-level amplitude variability and provides complementary information to spectral features.

Four feature modes were evaluated: PSD, DE, PSD+DE, and PSD+DE+LOGVAR. These feature groups were selected because spectral power [18], entropy-related representations [19], and signal variability have been widely used in EEG biometric recognition and authentication studies. PSD features are physiologically interpretable and computationally efficient, while DE and log-variance features provide complementary information about signal complexity and amplitude variability.

### 2.6. Models and Supplementary Analyses

Four classical classifiers were evaluated in the handcrafted feature branch: SVM, LDA, LR, and RF. Before classifier training, feature vectors were standardized using z-score normalization. The mean and standard deviation were estimated only from the training set and then applied to validation, calibration, and blind testing sets. Hyperparameters were selected based on validation-set accuracy. An EEGNet-like compact convolutional neural network was used as the raw EEG baseline, as shown in Figure 3b. The network consisted of temporal convolution, depthwise spatial convolution, a separable-style convolution block, and a fully connected classifier. CNN-based EEG models have been widely studied because convolutional structures can learn local temporal and spatial patterns from raw or weakly processed EEG signals [9,12].

The EEGNet-like baseline used event-level raw EEG segments as input. Each input sample had a size of 64×2000, corresponding to 64 channels and 4 s of EEG data at 500 Hz. The temporal convolution block used 8 filters with a kernel size of 1×64, followed by batch normalization. The depthwise spatial convolution block used 16 filters with a kernel size of 64×1 and 8 groups, followed by batch normalization, ELU activation, average pooling with a kernel size of 1×4, and dropout with a rate of 0.25. The separable-style convolution block used 16 filters with a kernel size of 1×16, followed by batch normalization, ELU activation, average pooling with a kernel size of 1×8, and dropout with a rate of 0.25. The flattened output was passed to a fully connected layer for subject classification. The model was trained using the Adam optimizer with a learning rate of $1\times 10^{-3}$ and cross-entropy loss. The batch size was 32, and the maximum number of training epochs was 30. No early stopping was applied; instead, the model was trained for all epochs, and the model state with the highest validation accuracy was retained for final blind testing. Feature ablation was performed to compare PSD, DE, PSD+DE, and PSD+DE+LOGVAR. Channel selection was performed using a one-way ANOVA F-score calculated exclusively from the training set. For channel *c* and feature dimension *d*, the F-score was defined as

(5) $$F_{c,d}=\frac{\frac{1}{M-1}\sum_{m=1}^{M}n_{m}{\left(\mu_{m,c,d}-\mu_{c,d}\right)}^{2}}{\frac{1}{N-M}\sum_{m=1}^{M}\sum_{i=1}^{n_{m}}{\left(x_{i,m,c,d}-\mu_{m,c,d}\right)}^{2}},$$

where *M* is the number of subject classes, $n_{m}$ is the number of training samples belonging to subject *m*, *N* is the total number of training samples, $\mu_{m,c,d}$ is the mean of feature dimension *d* for channel *c* in subject class *m*, and $\mu_{c,d}$ is the corresponding overall mean. Because each channel contained multiple feature dimensions, the final channel-ranking score was obtained by averaging the F-scores across all *D* feature dimensions:

(6) $$S_{c}=\frac{1}{D}\sum_{d=1}^{D}F_{c,d}.$$

Channels were ranked in descending order according to $S_{c}$, and the top-*k* subsets were evaluated for k=4, 8, 16, 32, and 64. Only training-set samples were used to calculate the channel ranking. Task robustness was evaluated using two strategies: single-task analysis, in which one task was used for training, calibration, and testing, and leave-one-task-out analysis, in which one task was held out as the blind testing condition while the remaining tasks were used for training and calibration.

### 2.7. Evaluation Metrics and Statistical Analysis

Closed-set identification was evaluated by treating subject identity as a multi-class classification target. Rank-1 accuracy was used as the primary metric. Rank-3, Rank-5, CMC curves, precision, recall, macro-F1, and subject-level F1-scores were also reported. Rank-*N* accuracy was defined as the proportion of blind testing samples for which the true identity appeared within the top-N predicted identities. Biometric verification was evaluated by converting multi-class identity scores into genuine and impostor scores. Identity scores were derived from classifier decision outputs rather than calibrated probabilities. ROC-based verification metrics were computed retrospectively by sweeping decision thresholds over the pooled genuine and impostor scores from the blind-test set. No single operational authentication threshold was selected or fixed for threshold-based classification of the blind-test samples. Accordingly, EER and TAR at fixed FAR levels should be interpreted as score-level separability measures rather than prospective fixed-threshold authentication performance. For each blind testing sample, the score assigned to the true identity was treated as the genuine score, while the scores assigned to all other identities were treated as impostor scores. Verification metrics included EER, ROC curves, AUC, TAR@FAR = 1%, and TAR@FAR = 0.1%. These metrics were included because practical biometric systems require low false-acceptance performance, and classification accuracy alone is insufficient to characterize authentication security [20]. To account for the dependence among multiple impostor scores derived from the same blind-test event, an additional subject-level one-versus-rest verification analysis was performed. For each enrolled participant, each blind-test event contributed one score corresponding to that claimed identity. Scores from events belonging to the same participant were treated as genuine scores, whereas scores from all other participants were treated as impostor scores. EER was calculated separately for each participant and each repeated run. The run-level EER values were then averaged within each participant, and a 95% confidence interval for the mean subject-level EER was estimated by resampling participants with replacement using 10,000 bootstrap iterations.

All experiments were repeated for 20 independent runs with different random seeds. Repeated runs with different random seeds were used to assess split-related variability. Because repeated random seeds are not independent biological replicates, run-level statistical tests were interpreted as algorithmic robustness analyses, whereas participant-level summaries were used for biological interpretation. Results are reported as mean ± standard deviation unless otherwise stated. Paired t-tests and Wilcoxon signed-rank tests were used to compare performance with and without calibration, with statistical significance set at p<0.05.

## 3. Results

### 3.1. Main Cross-Day Identification Performance and Calibration Benefit

**Figure 4:**
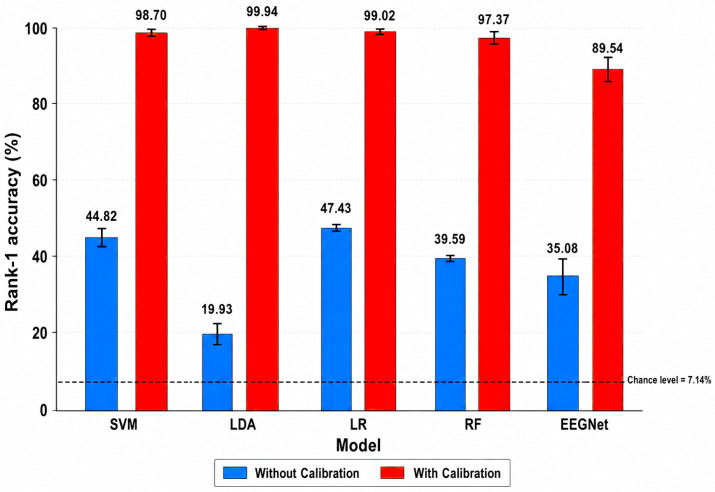
Cross-day Rank-1 identification performance under with- and without-calibration settings.

The main cross-day experiment evaluated whether target-session calibration could improve identity recognition under a trial-level non-overlapping data partitioning strategy. Session 0 was used for training and validation, while Session 1 was used for calibration and blind testing. Subject identity was treated as the classification target, and Rank-1 accuracy was used as the primary closed-set identification metric. As shown in Figure 4 and Table 2, all models in the without-calibration setting achieved above-chance performance but remained limited under direct cross-day testing. In the without-calibration setting, LR achieved the highest Rank-1 accuracy of 47.43 ± 0.81%, followed by SVM with 44.82 ± 1.23%, RF with 39.59 ± 0.74%, EEGNet with 35.08 ± 5.71%, and LDA with 19.93 ± 1.17%. These results indicate that subject-specific EEG signatures were partially preserved across days, but direct generalization was substantially affected by session-related distribution shifts.

After target-session calibration, blind Rank-1 accuracy increased markedly across all models. In the with-calibration setting, LDA achieved the highest Rank-1 accuracy of 99.94 ± 0.09%, followed by LR (99.02 ± 0.38%), SVM (98.70 ± 0.44%), RF (97.37 ± 0.74%), and EEGNet (89.54 ± 3.73%). The absolute Rank-1 improvement from the without-calibration setting to the with-calibration setting ranged from +51.59 percentage points for LR to +80.01 percentage points for LDA. In addition, Rank-3 and Rank-5 accuracies in the with-calibration setting approached saturation for most classical models, indicating that calibration improved not only top-1 prediction but also the ranking quality of identity scores. Statistical comparisons of Rank-1 accuracy between the settings without and with target-session calibration across 20 repeated runs are provided in Supplementary Table S1. After Holm–Bonferroni correction, all classical models showed significant improvements after target-session calibration, with large effect sizes.

**Figure 5:**
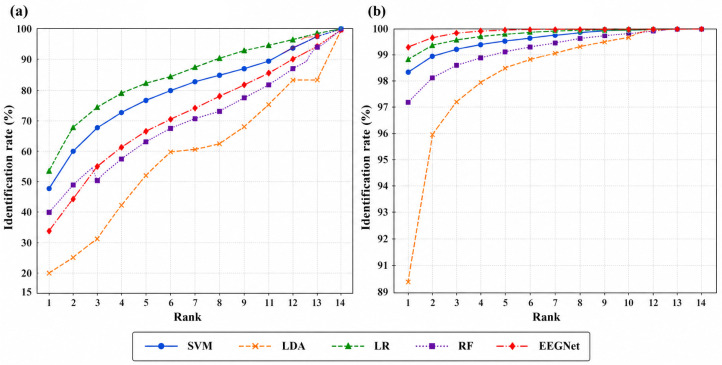
Cumulative match characteristic curves under with- (**a**) and without-calibration (**b**) settings.

The CMC curves in Figure 5 further confirmed the calibration benefit. In the no-calibration setting, identification rates increased gradually as the rank threshold increased, suggesting that the correct identity was often among the top candidates but not consistently ranked first. In contrast, the calibration setting produced near-saturated Rank-*N* performance at low ranks. Subject-level F1-score analysis in Supplementary Figure S1 showed that handcrafted feature-based classical models achieved consistently high F1-scores for most participants, whereas EEGNet exhibited larger between-subject variability. This suggests that the handcrafted branch provided more stable subject-level recognition under the current cross-day setting.

### 3.2. Calibration-Amount Analysis

**Figure 6:**
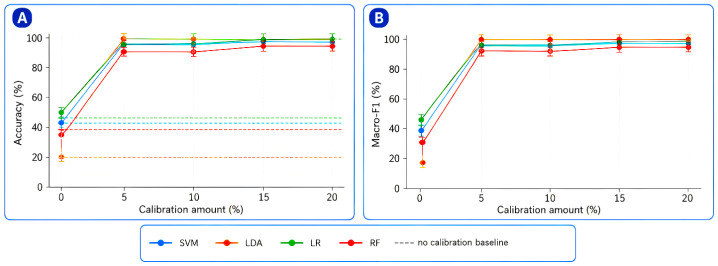
Effect of target-session calibration amount on cross-day EEG identity recognition. (**A**) accuracy and (**B**) macro-F1 were evaluated under 0%, 5%, 10%, 15%, and 20% target-session calibration ratios.

To investigate how much target-session data was required for effective adaptation, calibration ratios of 0%, 5%, 10%, 15%, and 20% were evaluated. Four classical classifiers were compared under the same trial-level non-overlapping data partitioning strategy. Rank-1 accuracy and macro-F1 were used to quantify the effect of increasing calibration data. As shown in Figure 6, all classifiers showed a sharp performance improvement when only 5% of target-session samples were used for calibration. In this calibration-amount analysis, the 0% condition was used as the baseline of the calibration curve, with no target-session samples reserved for calibration. Because the blind-test subset changed with the calibration ratio, the 0% values in this analysis were not numerically identical to the without-calibration values in Table 2, which were computed under the default 15% calibration split used for the main model comparison. At 0% calibration, Rank-1 accuracy ranged from 19.83 ± 1.10% for LDA to 47.17 ± 0.58% for LR. After introducing 5% target-session calibration, Rank-1 accuracy increased to 96.86 ± 1.41% for SVM, 99.92 ± 0.12% for LDA, 97.60 ± 0.45% for LR, and 94.61 ± 0.99% for RF. The macro-F1 results showed a similar trend, indicating that the calibration benefit was not driven by a small number of subjects but reflected balanced improvement across subjects.

Further increasing the calibration ratio from 5% to 10% produced little additional improvement for most models. When calibration was increased to 15% and 20%, SVM, LR, and RF showed modest gains, while LDA remained close to ceiling performance. At the default 15% calibration setting, Rank-1 accuracy reached 98.70 ± 0.44% for SVM, 99.94 ± 0.09% for LDA, 99.02 ± 0.38% for LR, and 97.37 ± 0.74% for RF, consistent with the main all-channel results reported in Table 2. At 20% calibration, Rank-1 accuracy reached approximately 98.80 ± 0.47% for SVM, 99.94 ± 0.09% for LDA, 99.05 ± 0.35% for LR, and 97.52 ± 0.66% for RF. These results indicate that most of the cross-session performance loss can be recovered with limited target-session data. Although 15% calibration was used as the default setting for stable model comparison, the 5% result suggests that practical calibration burden can be substantially reduced.

### 3.3. Biometric Verification Performance

**Figure 7:**
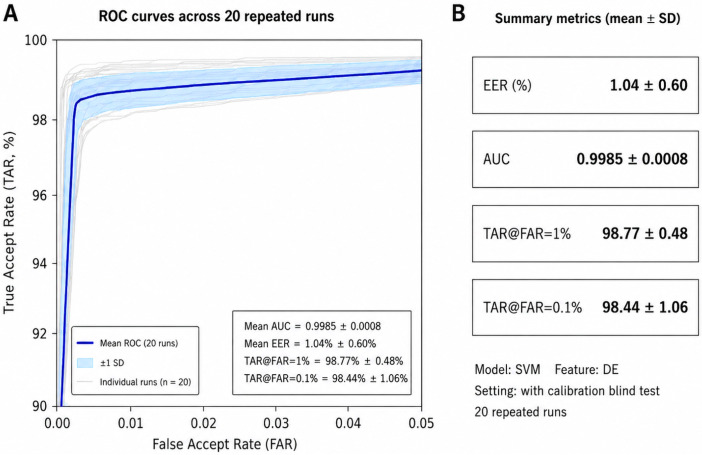
Biometric verification performance under the calibration setting. (**A**) ROC curves across 20 repeated runs. Gray curves indicate individual runs, the blue curve indicates the mean ROC, and the shaded region represents ±1 standard deviation. (**B**) Summary verification metrics, including EER, AUC, TAR@FAR = 1%, and TAR@FAR = 0.1%. The SVM classifier with DE features achieved low EER and high TAR at low FAR operating points, indicating strong genuine–impostor separability under cross-day calibration.

Beyond closed-set identification, we evaluated whether the proposed framework could support biometric verification. Multi-class identity scores were converted into genuine and impostor scores. For each blind testing sample, the score assigned to the true identity was treated as the genuine score, whereas scores assigned to other identities were treated as impostor scores. Verification performance was evaluated using ROC curves, EER, AUC, and TAR at fixed FAR values. As shown in Figure 7A, ROC curves from 20 repeated blind-test runs were consistently located near the upper-left corner, indicating strong genuine–impostor separability. The mean ROC curve rose steeply at very low FAR values, suggesting that most genuine samples could be accepted while maintaining a low false-acceptance rate. The narrow standard deviation band indicated stable verification performance across repeated runs.

Using SVM with DE features under the blind-test calibration setting, the model achieved an EER of 1.04±0.60% and an AUC of 0.9985±0.0008. In addition, TAR@FAR = 1% reached 98.77±0.48%, and TAR@FAR = 0.1% reached 98.44±1.06%. These results show that the learned EEG representations were not only discriminative for closed-set identity assignment but also effective for separating genuine and impostor trials under strict false-acceptance constraints. The genuine scores were 13.282±0.289, with a median of 13.310 [13.307–13.311], whereas the impostor scores were 5.941±3.930, with a median of 5.809 [2.725–9.235]. This separation between matched and non-matched identity scores supports the low EER, near-perfect AUC, and high TAR@FAR values observed in the verification analysis. Because multiple non-matching identity scores were generated from each blind-test event, we additionally evaluated verification performance at the participant level. The mean subject-level EER was 1.57 ± 2.05%, with a participant-clustered bootstrap 95% confidence interval of 0.63%–2.70%. The median subject-level EER was 0.34% [IQR: 0.01%–3.00%], with individual participant values ranging from 0% to 6.74%. These findings confirm generally low verification error while also revealing meaningful between-participant variability that is not reflected in the pooled score-level EER.

### 3.4. Feature Selection and Feature Contribution

**Figure 8:**
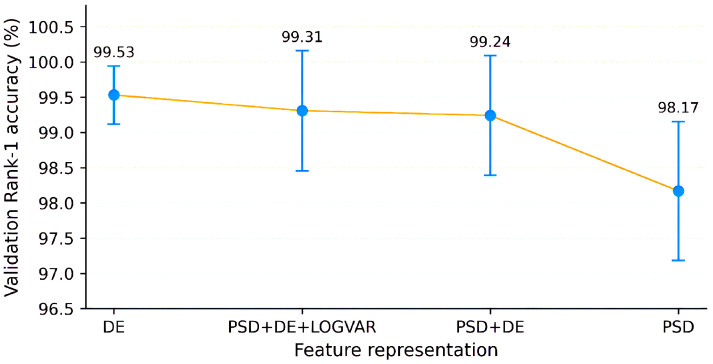
Feature selection based on SVM validation performance.

To determine a compact and effective handcrafted representation, four feature modes were compared using SVM validation performance: PSD, DE, PSD+DE, and PSD+DE+LOGVAR. Validation Rank-1 accuracy was used as the selection criterion because it reflects the discriminative capacity of each feature representation before final blind testing. As shown in Figure 8, all candidate features achieved high validation Rank-1 accuracy, indicating that the extracted spectral and statistical features contained strong subject-discriminative information. DE achieved the highest mean validation Rank-1 accuracy of 99.53%, followed by PSD+DE+LOGVAR (99.31%), PSD+DE (99.24%), and PSD (98.17%). However, the validation performance of DE, PSD+DE, and PSD+DE+LOGVAR was close to ceiling and numerically similar, indicating that the small differences among these feature representations should not be overinterpreted. Therefore, DE was selected as a compact representative handcrafted feature for subsequent biometric verification analysis, rather than being interpreted as clearly superior to the combined feature sets. The lower mean performance and larger variability of PSD suggest that relative spectral power alone may be less stable than DE under the current cross-day setting. The addition of PSD and LOGVAR to DE did not provide a clear validation-level improvement, suggesting possible feature redundancy under the present SVM-based setting. Overall, DE provided a compact and effective representation for calibrated EEG biometric recognition, although its advantage over combined feature sets should be interpreted cautiously given the near-ceiling validation performance.

### 3.5. Channel Selection and Deployment Feasibility

**Figure 9:**
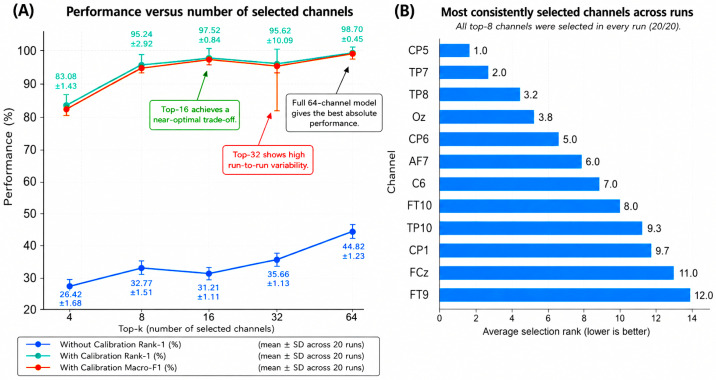
Top-k channel selection analysis for cross-day EEG biometric recognition. (**A**) Identification performance as a function of the number of selected channels. (**B**) Most consistently selected channels across runs, summarized by average selection rank (lower is better). All top-8 channels were selected in every run (20/20), indicating stable channel importance across repeated experiments.

To assess whether the framework could operate with fewer electrodes, top-k channel selection was performed using k = 4, 8, 16, 32, and 64. Channel ranking was derived only from the training set, and selected channel subsets were evaluated under the same cross-day protocol. As shown in Figure 9A, in the without-calibration setting, Rank-1 accuracy increased from 26.42 ± 1.68% with the top-4 channels to 44.82 ± 1.23% with all 64 channels, indicating that direct cross-day generalization benefits from broader spatial coverage. In the with-calibration setting, performance increased sharply from 83.08 ± 1.43% with top-4 channels to 95.24 ± 2.92% with top-8 channels and 97.52 ± 0.84% with top-16 channels. Beyond 16 channels, additional gains were small, although the full 64-channel configuration achieved the best Rank-1 performance of 98.70 ±0.44%.

This value corresponds to the Rank-1 standard deviation in the top-64 channel setting and is consistent with Table 2. The macro-F1 trend closely followed Rank-1 accuracy, suggesting that reduced-channel models maintained balanced subject-level recognition. Figure 9B summarizes the most frequently and consistently selected electrode positions in the main all-task setting. The leading electrodes were repeatedly ranked near the top across repeated runs, suggesting that some spatial locations contributed more strongly to subject discrimination than others. However, the exact top-ranked channels were not assumed to be invariant across tasks or data splits. Therefore, the top-k analysis should be interpreted as evidence for the feasibility of reducing electrode density under data-driven channel selection, rather than as proof of a universal fixed reduced-electrode montage. Overall, these results indicate that a compact top-16 configuration can provide a favorable trade-off between recognition performance and system complexity in the present calibrated cross-day setting, while the full-channel configuration remains preferable when maximum performance and robustness are required.

### 3.6. Task-Level Contribution and Cross-Task Robustness

**Figure 10:**
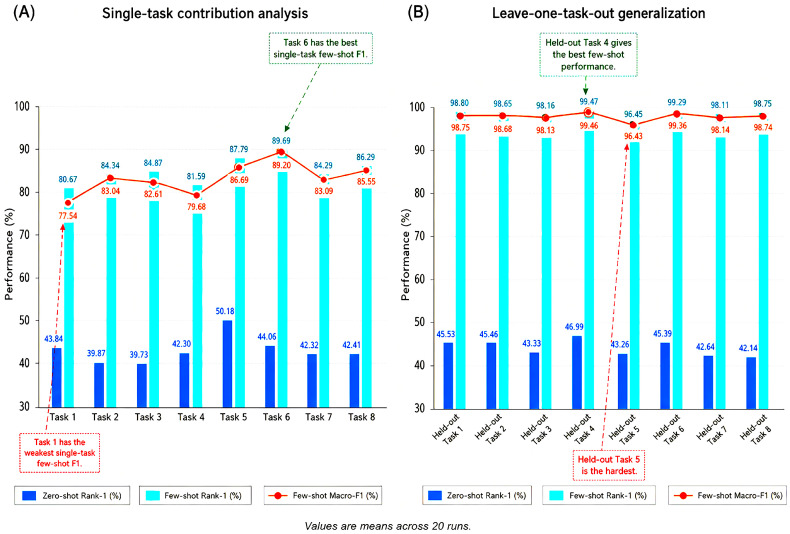
Task-level contribution and leave-one-task-out generalization analysis. (**A**) Single-task contribution analysis. (**B**) Leave-one-task-out generalization analysis. ZS, zero-shot setting without target-session calibration; FS, few-shot setting with target-session calibration.

Task-level analyses were performed to examine whether identity recognition was dominated by a specific motor-related task. In the single-task analysis, each task was independently used for training, calibration, and testing. In the leave-one-task-out analysis, one task was held out as the blind testing condition while the remaining tasks were used for training and calibration. As shown in Figure 10A, all single-task settings achieved higher performance in the with-calibration setting than in the without-calibration setting, indicating that each task contained useful identity-discriminative information. Rank-1 accuracy in the without-calibration setting ranged from 39.73% for Task 3 to 50.18% for Task 5.

After calibration, Rank-1 accuracy in the with-calibration setting ranged from 80.67% for Task 1 to 89.69% for Task 6, while macro-F1 ranged from 77.54% to 89.20%. These results indicate that all tasks contributed to identity recognition, although their discriminative strength differed. The leave-one-task-out results in Figure 10B further demonstrated cross-task robustness. When each task was held out for blind testing, Rank-1 accuracy in the without-calibration setting remained around 42–47%. After calibration, Rank-1 accuracy in the with-calibration setting remained consistently high across held-out tasks, ranging from 96.45% for held-out Task 5 to 99.47% for held-out Task 4. Macro-F1 showed the same trend. These results suggest that the learned identity representation was not restricted to a single task condition but generalized across motor-related tasks.

### 3.7. Validation on External Public Multi-Session MI Dataset

**Figure 11:**
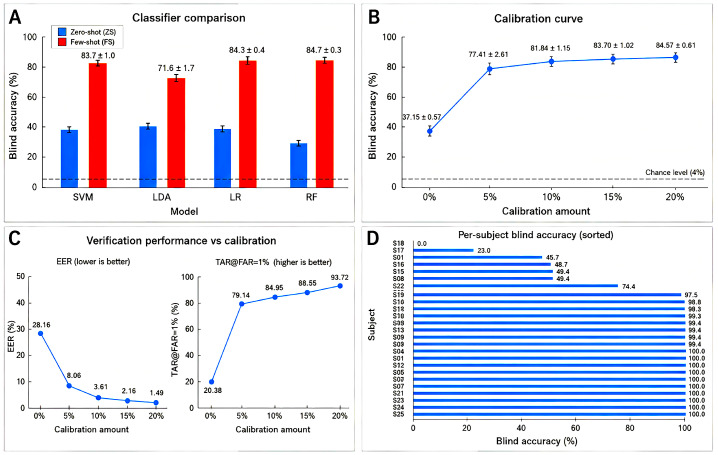
Validation on external public multi-session MI dataset. (**A**) Classifier comparison under zero-shot and few-shot settings. The dashed line indicates the chance level of 4%. (**B**) Calibration curve showing blind accuracy under 0%, 5%, 10%, 15%, and 20% target-session calibration. (**C**) Verification performance as a function of calibration amount, including EER and TAR@FAR = 1%. (**D**) Sorted per-subject blind accuracy under the calibration setting. ZS, zero-shot setting without target-session calibration; FS, few-shot setting with target-session calibration.

To examine generalizability beyond the in-house dataset, the same pipeline was applied to the external public multi-session MI dataset. Sessions 1–3 were used as source sessions, while Sessions 4–5 were used as target sessions for calibration and blind testing. Subject identity was used as the recognition target, and left/right MI labels were treated as task conditions. As shown in Figure 11A, external public datasets results showed the same general pattern as the in-house dataset. Performance without calibration was above the 4% chance level, but few calibration produced large improvements. With calibration, SVM, LDA, LR, and RF achieved blind accuracies of 83.7 ± 1.0%, 71.6 ± 1.7%, 84.3 ± 0.4%, and 84.7 ± 0.3%, respectively. The calibration curve in Figure 11B showed that blind accuracy improved from 37.15 ± 0.57% without calibration to 77.41 ± 2.61% with only 5% calibration. Accuracy further increased to 81.84 ± 1.15%, 83.70 ± 1.02%, and 84.57 ± 0.61% at 10%, 15%, and 20% calibration, respectively. Verification metrics also improved with calibration. As shown in Figure 11C, EER decreased from 28.16% without calibration to 8.06%, 3.61%, 2.16%, and 1.49% at 5%, 10%, 15%, and 20% calibration, respectively. TAR@FAR = 1% increased from 20.38% to 79.14%, 84.95%, 88.55%, and 93.72%. Subject-level accuracy in Figure 11D showed high performance for most subjects, although several subjects remained difficult to identify, indicating inter-subject variability in EEG biometric stability.

**Figure 12:**
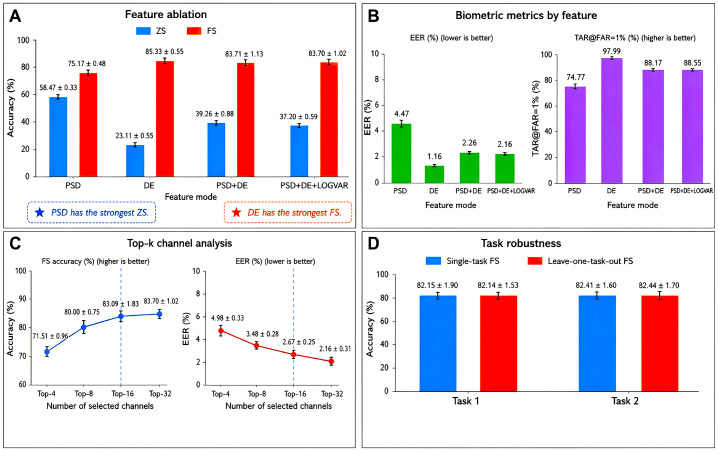
Ablation and robustness analysis on the dataset. (**A**) Feature ablation under without and with calibration settings. (**B**) Biometric metrics by feature representation, including EER and TAR@FAR = 1%. (**C**) Top-*k* channel analysis showing calibration accuracy and EER as a function of the number of selected channels. (**D**) Task robustness analysis comparing single-task and leave-one-task-out calibration performance across the two MI task conditions. ZS, zero-shot setting without target-session calibration; FS, few-shot setting with target-session calibration.

Additional robustness analyses are shown in Figure 12. Feature ablation showed that PSD achieved the strongest accuracy (58.47 ± 0.33%) without calibration, whereas DE achieved the strongest accuracy (85.33 ± 0.55%) with calibration. DE also achieved the lowest EER (1.16%) and highest TAR@FAR = 1% (97.99%). Top-k channel analysis showed that performance approached saturation around 16 channels. Task robustness analysis showed stable performance across the two MI conditions. These findings indicate that calibration efficiency, verification capability, reduced-channel feasibility, and task robustness were retained in the external dataset.

## 4. Discussion

This study demonstrates that cross-session EEG biometric identification and verification can be substantially improved by combining strict event-level evaluation, interpretable spectral features, and few-shot target-session calibration. The without-calibration results showed that subject-discriminative EEG information was partially preserved across recording days, but direct cross-day generalization remained limited. After incorporating a small amount of target-session calibration data, Rank-1 accuracy, CMC performance, macro-F1, and verification metrics all improved markedly. This finding supports the view that EEG biometrics should not be treated as a static classification problem, but as a non-stationary biometric signal processing problem. It is also consistent with recent EEG biometric studies emphasizing cross-session robustness, domain adaptation, graph-based modeling, and session-invariant feature learning [11,14]. A key methodological strength of this work is strict event-level isolation, in which all windows derived from the same marker-defined EEG event were kept within a single data subset, thereby preventing overlap among training, validation, calibration, and blind testing subsets. In EEG biometric studies, high performance obtained under within-session or randomly shuffled protocols may be inflated by window-level dependency, session-specific noise, electrode contact conditions, or temporal proximity between training and testing samples. By constructing one trial-level sample from each EEG trial and preventing trial overlap across subsets, the present framework reduces optimistic bias caused by window- or trial-level overlap and provides a more conservative estimate of cross-day performance within the present closed-set, calibration-assisted experimental protocol. The marked difference between the no-calibration and cohort-wide supervised recalibration settings further indicates that cross-session distribution shift is substantial and should be explicitly considered in EEG biometric evaluation.

The calibration-amount analysis suggests that target-session calibration is a practical and efficient adaptation strategy. The largest improvement occurred when the calibration ratio increased from 0% to 5%, whereas increasing calibration to 15–20% produced only modest additional gains. This finding is important because calibration time is a major barrier to real-world EEG biometric deployment. Although 15% calibration was used as the default setting to obtain stable model comparisons, the 5% result indicates that short target-session enrollment may be sufficient when a small performance trade-off is acceptable. Compared with more complex domain-adaptation frameworks [21,22], this calibration strategy is simple, transparent, and easy to implement. The feature analyses showed that DE was a compact and effective representation for calibrated EEG biometric recognition, while PSD provided relatively strong performance without calibration in the external public validation. PSD has long been used in EEG biometrics because it is computationally simple and physiologically interpretable [20,23]. DE may provide complementary entropy-related information about band-power distributions and signal complexity, which may explain its strong performance after calibration. The fact that adding PSD and LOGVAR to DE did not consistently improve validation performance suggests that increasing feature dimensionality does not necessarily improve cross-session identity recognition. For small and moderate EEG datasets, lightweight handcrafted features may provide a favorable balance among accuracy, interpretability, computational cost, and robustness.

The comparison between classical models and EEGNet also provides useful insight. Although deep learning methods have been widely adopted in EEG biometrics [1,12,13], the handcrafted feature branch achieved stronger and more stable performance in the present experiments. This does not imply that handcrafted features are universally superior to deep learning. Rather, it suggests that, under limited cross-session EEG data, protocol rigor, feature interpretability, and calibration design may be as important as model complexity. Future studies may combine interpretable spectral features with CNNs, Transformers, graph neural networks, Siamese networks, or triplet-loss learning to improve scalability and open-set recognition [24]. The verification results extend the findings beyond closed-set identification. Rank-1 accuracy and CMC curves evaluate whether the system can correctly assign an EEG sample to an enrolled subject, whereas practical authentication requires security-oriented metrics such as EER, FAR, FRR, ROC, and TAR at fixed FAR levels [20]. The low EER, near-perfect AUC, and high TAR@FAR values indicate that the learned EEG representations effectively separated genuine and impostor scores under the calibration setting. This distinction is important because high multi-class accuracy does not necessarily guarantee reliable authentication at low false-acceptance rates. Closed-set identification assumes that each test sample belongs to one of the enrolled subjects, while score-based verification evaluates genuine–impostor separability using identity scores. However, true authentication systems also require open-set impostor rejection, unknown-user detection, template protection, adversarial robustness, and deployment security evaluation. These aspects were beyond the scope of the present study and should be addressed in future work using dedicated open-set protocols and protected biometric template designs.

Channel and task analyses further support practical deployment. The top-16 channel subset preserved near-optimal performance with calibration, suggesting that a reduced electrode configuration can retain most identity-discriminative information. This finding is consistent with previous studies showing that reduced-channel EEG systems can retain biometric utility [25]. Task-level analyses showed that identity information was present across multiple motor-related conditions and generalized well in the leave-one-task-out setting. This suggests that the model did not simply rely on a single task-specific pattern. Nevertheless, task design influenced performance, consistent with reports that task state can modulate EEG identity information [26,27].

External validation provides additional evidence that the proposed framework is not specific to the in-house dataset. The same general pattern was observed: above-chance performance without target-session calibration, large gains after target-session calibration, improved verification metrics, reduced-channel feasibility, and task robustness. Public multi-subject and multi-session EEG datasets are important for assessing whether EEG biometric methods generalize beyond a single acquisition protocol [28,29]. However, subject-level results in external datasets also revealed inter-subject variability, indicating that some users may require adaptive enrollment, user-specific calibration amounts, or confidence-based rejection mechanisms. Similar user-dependent variability has been reported in EEG biometric studies [30]. The lower accuracy observed in this subset is likely related to user-dependent cross-session variability in motor imagery EEG. The external dataset contains multiple independent sessions, and some subjects may have larger session-related shifts due to differences in electrode placement, impedance, fatigue, attention state, signal quality, or motor imagery consistency. For these subjects, the limited target-session calibration samples may not sufficiently represent the target-session distribution, resulting in incomplete compensation for cross-session variability. We therefore interpret these cases as lower-performing subjects with unstable cross-session EEG identity patterns rather than complete identification failures.

Several limitations should be noted. First, the in-house dataset included two sessions per subject, so longer-term longitudinal data are needed to evaluate permanence over weeks or months. Second, although verification metrics were derived from genuine and impostor scores, the current framework remains primarily closed-set; future studies should examine open-set recognition, unknown-user rejection, and metric-learning approaches such as Siamese or triplet-loss models. Third, reduced-channel feasibility was evaluated analytically but not tested directly on dry electrodes, wearable headsets, or ear-EEG devices. Further validation on larger datasets and task-specific deployment protocols is needed before defining a fixed reduced-electrode montage. Fourth, this study focused mainly on spectral and entropy-related features; functional connectivity, Riemannian covariance, graph-based features, and spatial–spectral–temporal representations may provide complementary information about stable subject-specific brain networks. Finally, EEG biometric systems raise privacy and security concerns because neural signals may contain sensitive identity and cognitive information. Future work should consider cancelable templates, privacy-preserving representation learning, adversarial robustness, and identity-protection mechanisms.

## 5. Conclusions

This study proposed a cross-session EEG biometric identification and verification framework based on interpretable spectral features and target-session calibration. By using a trial-level non-overlapping data partitioning strategy, the framework provided a realistic evaluation of cross-day identity recognition by ensuring that all windows derived from the same EEG trial remained within the same data subset. Target-session calibration substantially improved Rank-1 accuracy, macro-F1, CMC performance, and biometric verification metrics. Calibration-amount analysis showed that only a small amount of target-session data was needed to recover most of the cross-session performance loss. DE features achieved strong validation and verification performance, while reduced-channel and task analyses supported practical deployment feasibility. External validation on the multi-session MI dataset confirmed that the proposed framework was not limited to the in-house dataset. Overall, these findings suggest that practical EEG biometric systems should be developed as strictly partitioned, calibration-efficient, and verification-aware frameworks rather than simple within-session classifiers.

## Figures and Tables

**Table 1:** Summary of the in-house EEG dataset and event-level sample allocation in the main cross-session experiment.

Item	Description
Number of participants	14
Closed-set chance level	7.14%
Recording sessions	Two independent sessions acquired on separate days
EEG channels	64 scalp channels
Sampling rate	500 Hz
Task conditions	8 motor-related task conditions
Total valid event-level samples	2225
Total source-session samples	1108
Total target-session samples	1117
Total training samples per run	884
Total validation samples per run	224
Total calibration samples per run	217
Total blind testing samples per run	900
Number of repeated runs	20

**Table 2:** Main cross-day identification performance.

Model	ZS Rank-1 (%)	FS Rank-1 (%)	FS Rank-3 (%)	FS Rank-5 (%)	FS Macro-F1 (%)	Gain (pp)
SVM	44.82 ± 1.23	98.70 ± 0.44	99.67 ± 0.18	99.87 ± 0.14	98.74 ± 0.45	53.88 ± 1.39
LDA	19.93 ± 1.17	99.94 ± 0.09	100.00 ± 0.00	100.00 ± 0.00	99.94 ± 0.09	80.01 ± 1.17
LR	47.43 ± 0.81	99.02 ± 0.38	99.89 ± 0.11	100.00 ± 0.00	99.02 ± 0.38	51.59 ± 0.65
RF	39.59 ± 0.74	97.37 ± 0.74	99.61 ± 0.23	99.87 ± 0.06	97.34 ± 0.77	57.78 ± 1.02
EEGNet	35.08 ± 5.71	89.54 ± 3.73	98.01 ± 1.54	99.42 ± 0.65	89.17 ± 3.97	54.46 ± 5.91

ZS, zero-shot setting without target-session calibration; FS, few-shot setting with 15% target-session calibration. All performance values are percentages and are reported as mean ± standard deviation across repeated runs. Gain denotes the percentage-point improvement in FS Rank-1 over ZS Rank-1. For classical models, the selected feature mode was DE; EEGNet used raw EEG input.

## Data Availability

The SHU dataset used for external validation is publicly available. The in-house EEG dataset is not publicly available due to ethical and privacy restrictions, but can be made available from the corresponding author upon reasonable request.

## Supplementary Materials

The following supporting information can be downloaded at https://www.mdpi.com/article/10.3390/s26154811/s1, Table S1: Comparison of Rank-1 identification accuracy between the no-calibration and calibration-assisted settings across 20 repeated runs.

## Abbreviations

The following abbreviations are used in this manuscript: AUC, area under the receiver operating characteristic curve; BCI, brain–computer interface; CMC, cumulative match characteristic; DE, differential entropy; EEG, electroencephalography; EER, equal error rate; FAR, false accept rate; FRR, false reject rate; LDA, linear discriminant analysis; LR, logistic regression; MI, motor imagery; PSD, power spectral density; RF, random forest; ROC, receiver operating characteristic; SVM, support vector machine; TAR, true accept rate.
