# Supplementary material for: Interpretable Spectral Features for Cross-Session EEG Biometric Identification and Verification
Source: Sensors (Basel). 2026 Jul 29;26(15):4811. doi: 10.3390/s26154811 (PMC13469576; doi:10.3390/s26154811)
Supplement: Supplementary file 1 [file sensors-26-04811-s001.zip › sensors-4413785-supplementary.pdf]

Table S1 Comparison of Rank-1 identification accuracy between the no-calibration and calibration-assisted settings across 20 repeated runs

Model	ZS Rank-1 (%)	FS Rank-1 (%)	Mean difference (pp)	t	p_t	Holm p_t	Wilcoxon W	p_W	Holm p_W	Cohen dz
SVM	44.82 ± 1.26	98.70 ± 0.46	53.88	169.18	1.16E-31	1.16E-31	0	8.83E-05	3.42E-04	37.83
LDA	19.93 ± 1.20	99.94 ± 0.09	80.01	298.34	2.42E-36	7.27E-36	0	8.78E-05	3.42E-04	66.71
LR	47.43 ± 0.83	99.02 ± 0.39	51.59	345.01	1.53E-37	6.13E-37	0	8.56E-05	3.42E-04	77.15
RF	39.59 ± 0.76	97.37 ± 0.76	57.78	245.71	9.68E-35	1.94E-34	0	8.82E-05	3.42E-04	54.94

ZS, zero-shot setting without target-session calibration; FS, few-shot setting with target-session calibration
